# Study on frequency of dental developmental alterations in a Mexican school-based population

**DOI:** 10.4317/medoral.20691

**Published:** 2016-03-06

**Authors:** Constantino Ledesma-Montes, Maricela Garcés-Ortíz, Juan-Francisco Salcido-García, Florentino Hernández-Flores

**Affiliations:** 1 Clinical Oral Pathology Laboratory. División de Estudios de Posgrado e Investigación. Facultad de Odontología, Universidad Nacional Autónoma de México. México, 04510, D.F. MÉXICO; 2 Oral Diagnosis Clinic. División de Estudios de Posgrado e Investigación. Facultad de Odontología, Universidad Nacional Autónoma de México. México, 04510, D.F. MÉXICO; 3 Oral and Maxillofacial Surgery Clinic. División de Estudios de Posgrado e Investigación. Facultad de Odontología, Universidad Nacional Autónoma de México. México, 04510, D.F. MÉXICO

## Abstract

**Background:**

The aim of this study was to know the distribution of dental developmental alterations in the population requesting stomatological attention at the Admission and Diagnosis Clinic of our institution in Mexico City.

**Material and Methods:**

We reviewed the archives and selected those files with developmental dental alterations. Analyzed data were diagnoses, age, gender, location and number of involved teeth.

**Results:**

Of the 3.522 patients reviewed, 179 (5.1%) harbored 394 developmental dental alterations. Of them, 45.2% were males and 54.8% were females with a mean age of 16.7 years. The most common were supernumeraries, dental agenesia and dilaceration. Adults were 30.7% of the patients with dental developmental alterations. In them, the most common lesions were agenesia and supernumeraries. Mesiodens was the most frequently found supernumerary teeth (14.7%).

**Conclusions:**

Our finding that 30.7% of the affected patients were adults is an undescribed and unusually high proportion of patients that have implications on planning and prognosis of their stomatological treatment.

**Key words:**Developmental dental alterations, developmental alterations, supernumerary teeth, dental agenesia, root dilaceration.

## Introduction

Frequency of developmental dental alterations (DDAs) have been analyzed and reported. Most of these articles dealt on data from selected populations, entities, ethnic groups, countries, ages and genders using clinical, radiographic or clinico-radiographic approaches. During many years, the clinico-radiographic and radiographic studies employed different imagenologic techniques and the most common were orthopantomograms; some studies complemented their data with dento-alveolar radiographs.

Manuscripts were published with different titles and headings, analyzing the results from case series obtained from diverse populations, as children of specific age groups (1,2), orthodontic patients (3-5) and children under different clinical conditions (6,7). Some controlled studies examined a group of dental alterations (4,5,7-15), alterations in syndromic or non-syndromic patients (4,9,12,16). Also, some of them studied one (2,9,11-14) or two entities (4,16) in a group of patients. Other studies compared the frequency of different alterations among two different populations (11) or studied an isolated ethnic group (15,17). Unfortunately, this diversity of methods employed to design the patients sample did not demonstrate the authentic frequency of the pathologic entities in the general population. There are few studies in populations attending health or dental services with protocols including radiographic material for diagnosis analyzing patients grouped in more than five decades (17-19).

In view that many published manuscripts evaluating diverse populations, and their authors used different inclusion criteria and methods, we decided to design our study including all dental alterations in all patients attending our institution.

The aim of this study was to know the distribution of DDAs in the population requesting stomatological attention at the Admission and Diagnosis Clinic of our institution in Mexico City.

## Material and Methods

This study included all patients who sought stomatological attention during one year in the Admission and Diagnosis Clinic, Facultad de Odontología, UNAM. All patients and parents signed a Letter of Consent giving permission to use data for research purposes and the Ethics Committee approved the study. At first appointment, all the patients received an oral and maxillofacial examination. This assessment included careful observation and palpation of the soft and hard oral tissues and careful review of the head and neck area. A panoramic radiograph was made to all patients and all radiographs were reviewed and discussed by the panel. All discrepancies were solved by consensus and agreement.

Documented data were age, gender, diagnosis, location and affected teeth or tooth and findings were recorded in specially designed forms. Data on developmental alterations in third molars were not included in the study.

## Results

Out of the 3,522 patients. 179 (5.1%) presented one or more DDAs. These patients harbored 394 cases comprising 18 entities and their ages varied from 2 to 78 years. Main data is in  Table 1.

**Table 1:**
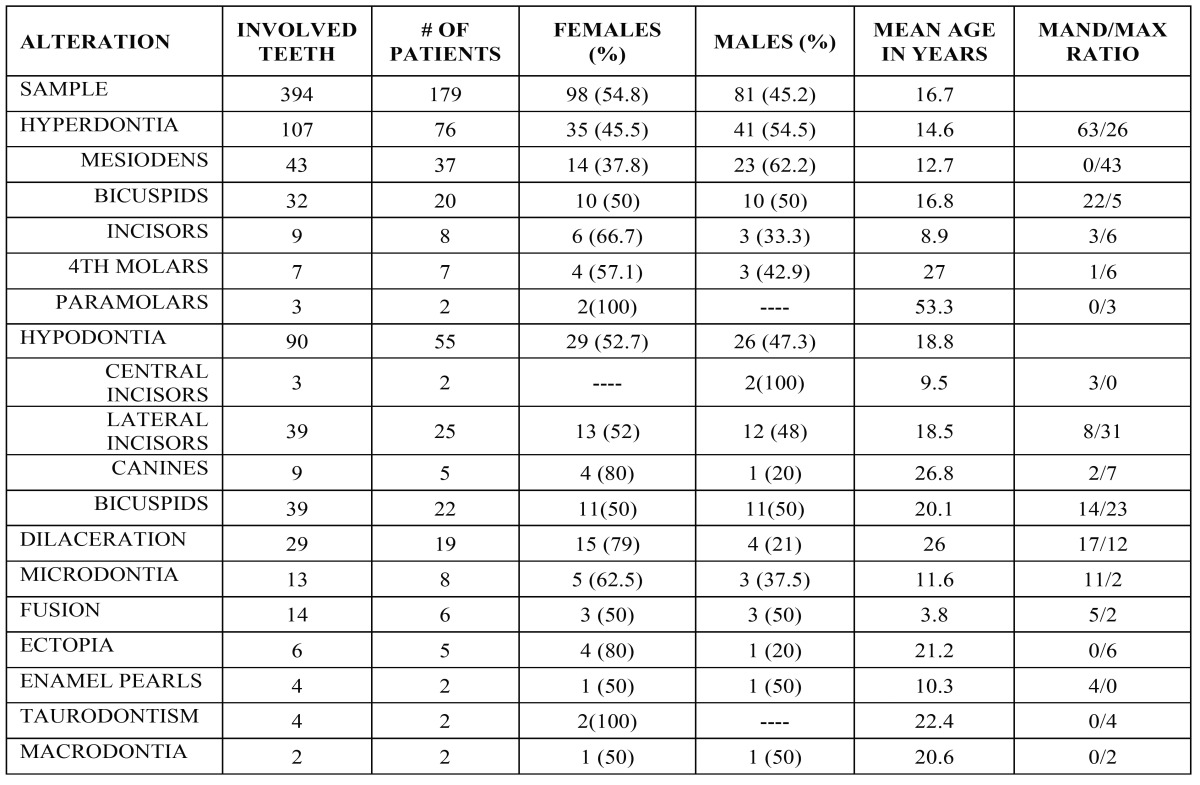
Frequency of dental developmental anomalies in the analized simple.

- Hyperdontia. Supernumerary teeth comprised 27.2% of the DDAs.

Mesiodens. They were 1.2% of the attended population and 10.9% of the DDAs and patients’ age varied from 2 to 55 years. There were 23 unique cases (53.5%), 5 patients presented 2 mesiodens (n= 10; 23.3%), 8 cases were in an inverted position (18.6%) and 2 mesiodens were found unerupted and fused with other teeth (4.6%). 

Supernumerary bicuspids were 8.1% of the DDAs. Nineteen bicuspids were in men (59.4%) and 13 teeth in women (40.6%) with ages fluctuating from 3 to 30 years. 22 supernumerary bicuspids were located in mandible (68.8%) and 10 were maxillary teeth (31.2%). There were 12 patients with one supernumerary tooth (60%), four presented two supernumeraries each (20%) and four cases with three bicuspids were found (20%).

Supernumerary incisors comprised 2.3% of the DDAs and age of the patients were between 4 and 24 years. Eight of them were from the permanent dentition (88.9%) and one was a deciduous tooth (11.1%). In addition, six were seen in maxilla (66.7%) and three were located in mandible (33.3%).

Fourth molars were 1.8% of the evaluated alterations and age of the patients varied from 12 to 41 years Six teeth were found in maxilla (85.7%) and one in mandible (14.3%).

Paramolars were maxillary teeth in two females, representing 0.8% of the assessed alterations.

Interestingly, one female patient harbored 12 supernumerary teeth in three quadrants. These teeth were one right upper canine; one left mandibular molar; three left mandibular bicuspids, one left mandibular canine; one right mandibular lateral incisor; one right mandibular canine; three right mandibular bicuspids and one right mandibular molar. In addition, one lower supernumerary canine was located in one boy.

- Hypodontia. This group comprised 25.9% of the patients with DDAs and recorded cases were 22.8% of the dental developmental cases. Age of the involved patients were from 3 to 78 years. Of the 90 missing teeth: 87 belonged from the permanent dentition and three were primary teeth (95.8% and 4.2% respectively). In the permanent dentition, the most frequently missing teeth was lateral incisor followed by second bicuspids (n=29; 33.3%) and first bicuspids (n= 10; 11.5%). Congenitally missing deciduous teeth were three mandibular cases.

- Dilaceration. This entity was in 0.5% of the attended population and affected teeth were 7.4% of the dental developmental alterations Patients’ age varied from 9 to 52 years and the most frequently affected teeth were bicuspids (n= 13; 44.8%) followed by molars (n= 8, 27.6%).

- Microdontia. Microdontic teeth signified 3.3% of the DDAs and affected patients’ age was between 5 and 36 years. The most frequent microdont was permanent lateral incisor (n= 9; 69.2%) followed by bicuspids (n= 3; 23.1%) and one deciduous lateral incisor.

- Fused Teeth. This group represented 8% of the DDAs and patients’ age varied from two to ten years. Five cases were fused primary teeth and two were in permanent dentition. Interestingly, one of the cases of the permanent dentition was associated to one supernumerary lateral incisor. All cases appeared in the anterior zone, two were maxillary cases (25%) and five were mandibular examples (75%).

- Dentinogenesis Imperfecta. This entity was represented by 80 teeth and consisted of 20.3% of the dental developmental alterations Patients with dentinogenesis imperfecta were two women and one man; both women were 30 years old and all permanent teeth (from 17 to 27 and from 37 to 47) were affected (28 teeth each). The man was a boy, son of one of the examined women. He presented twenty deciduous teeth and four first permanent molars involved.

- Amelogenesis Imperfecta. It was found in three boys and one girl comprising 2.2% of the analyzed patients and 13.2% of the DDAs. Patients’ age was 6 and 5 years (mean age= 10 years). One of the boys presented the generalized type of amelogenesis imperfecta including deciduous canine and both molars of the four quadrants (12 teeth). In addition, all his permanent dentition (from central incisor to second molar) was affected (28 teeth). Affected teeth in other two patients were two mandibular second bicuspids, one permanent upper lateral incisor and in the remaining child, involved teeth were two mandibular second bicuspids.

- Ectopic Teeth. They were three canines, two bicuspids and one central incisor (50%, 33.3% and 16.7% respectively) comprising 1.4% of the developmental alterations and 1.5% of the DDAs.

- Enamel Pearls. They were found in four deciduous mandibular molars (two first and two second molars). Frequency in this study was 1%.

- Taurodontism. It was observed in first molars only and frequency was 1%.

- Macrodontia. It was observed in one canine and one central incisor. In addition, we detected one supernumerary root in a permanent lower right first bicuspid.

Adult patients were 55 persons. They were 30.7% of the analyzed sample with DDAs. The most common entities were agenesia (n= 27 teeth; 35.8%) followed by supernumeraries (34.7%) and dilaceration (23.1%). The most commonly missing teeth were maxillary lateral incisors and bicuspids (n= 7; 7.4 each) that were 7.4% of the DDAs respectively. Mesiodens was the most frequently found supernumerary (14 teeth) representing 14.7% of the supernumeraries.

## Discussion

Agenesia was the more frequent entity we found in our population. In the literature, its prevalence varied from 0.3% to 36.5% (20) and the frequency we obtained was within this range. In our study, missing teeth were more common in females, permanent dentition was involved more frequently and the more commonly missing teeth were mandibular bicuspids, followed by maxillary lateral incisors. Data obtained in the studied population was in agreement with other reports (2,3-5,16-18,20-22). We found that the more commonly found missing teeth were lateral incisors followed by 2nd and first bicuspids. This finding agrees with results from other studies (1,3,5,6,15). Also, in our population, females missed teeth more frequently than males and others agree with us (3,5,15), but other studies reported that males were more commonly affected (1,17).

Our figure on the frequency of supernumerary teeth in the studied population was lower than those published in other reports. In these studies, frequency varied among 1.1% (16) to 2% (23). As it was communicated in many published studies (1,7,13-16,18), in our analysis we observed that supernumerary teeth were more frequent in males. This finding contrasts with those from other reports (6,19). In this study, mesiodens was the more frequently identified supernumerary tooth followed by bicuspids and fourth molars. This finding agrees with data from other studies (3,12,19). Also, some reports dealing on frequency of supernumeraries informed that fourth molars (10,11), bicuspids (16) or both fourth molars and mesiodens (22) were the most commonly found supernumeraries. Mesiodens prevalence in the attended population was 1.2% and comprised 10.9% of the DDAs. In other series, this prevalence varied from 0.4% in Finnish and 0.3 to 5.3% in Turkish populations (13,21).

Our findings reveal that the most common DDAs were missing teeth followed by supernumeraries in a similar rate. This finding seems to be unique to the population studied since other authors reported that frequencies for both entities were different (1,3,6,17,19). The presence of supernumeraries and missing teeth should be early detected since both entities produces occlusion problems and in these cases, orthodontic treatment is mandatory.

We found that frequency of microdontia was among 1.0% and 5.3% (3,4,6,17-19,22) and data obtained from the studied population is between both mentioned figures. In this study and according to most of the published reports (1,3,17), the more frequent microdontic teeth were maxillary lateral incisors. There is no complete agreement on the gender preference of this dental alteration (19-22), but in our study it was more common in females.

The frequency for dilaceration in previously reported studies was higher than that obtained by us. To date, the frequency we found is the lowest reported in the literature and is close to that obtained by Thongudomporn *et al.* in Australian population (3). In other studies it was between 9.5% and 15% (14,22).

Fused teeth is a rarely reported DDA more frequent in central and lateral incisors. In our study, it was found in mesiodens and in anterior maxillary and mandibular teeth. Our frequency of fused teeth of 8% of the DDAs is the highest reported to date.

Ectopic teeth also known as transposition was considered as one teeth occupying the position of another teeth or one teeth located outside its normal position. It is an uncommon DDA that should not be confused with hypo-hyperdontia. As in other studies (3,17), in ours the more commonly involved teeth were canines and bicuspids. It is considered that frequency is as low as 0.7% (19) and not higher than 14.4% (3).

Enamel pearls are a well-known but rarely reported DDAs more commonly observed in molars and is very frequency is wide among 0.22% to 33% (23,24). This data agrees with our results. In our investigation, taurodontism were within the reported frequency of 0.4% (19) to 46.4% (25).

It is interesting to note that dentinogesis and amelogenesis imperfecta comprised 170 teeth in seven patients. By number of involved teeth, they occupied the 3rd and 4th place respectively but by number of affected patients, they were in the last positions.

An unusual discovery in this study was the finding of a girl with multiple (26) supernumerary teeth. Our search in other family members discovered no affected relatives. Also, the patient showed no other developmental alterations and no association with any syndrome was found.

Early detection of intraoral DDAs is one of the objectives during the oral and maxillofacial review and this can be accomplished by means of the radiographic analysis, allowing to minimize stomatological complications associated with them.

In our study, we found an undescribed and unusually high proportion of adults affected by DDAs (30.7%). Figures from this study show that supernumeraries are present in a very wide age span, suggesting that the possibility to have serious interference during the stomatologic treatment already exists. Also, these figures call attention on the importance of the early detection of entities potentially harmful for the patient health and point to the need of a careful review of the panoramic radiographs before initiation any kind of treatment. This last assertion increases its importance in view that most of the orthodontic and prosthetic treatments are in the anterior area for aesthetic reasons. According to our results, the most common supernumerary teeth was mesiodens and there is general agreement that supernumeraries and missing teeth should be early detected since both entities could interfere with treatment (22). Various complications might occur as a result of the presence of supernumerary teeth, including cystic lesions, intraoral infection, rotation, root resorption of the adjacent teeth or even eruption of incisors in the nasal cavity and there are reports on the presence of odontogenic tumors associated with mesiodens (27,28).

## References

[1] Baccetti TA (1998). Controlled study of associated dental anomalies. Angle Orthod.

[2] Shabzendedar M, Mehrjerdia M (2010). Prevalence of hypodontia in nine-to fourteen-year-old children who attended the Mashhad School of Dentistry. Indian J Dent Res.

[3] Thongudomporn U, Freer TJ (1998). Prevalence of dental anomalies in orthodontic patients. Aust Dent J.

[4] Varela M, Arrieta P, Ventureira C (2009). Non-syndromic concomitant hypodontia and supernumerary teeth in an orthodontic population. Eur J Orthod.

[5] Gomes RR, da Fonseca JAC, Paula LM, Faber J, Acevedo AC (2010). Prevalence of hypodontia in orthodontic patients in Brasilia, Brazil. Eur J Orthod.

[6] Kathariya MD, Nikam AP, Chopra K, Patil NN, Raheja H, Kathariya R (2013). Prevalence of dental anomalies among school going children in India. J Int Oral Health.

[7] Grimanis GA, Kyriakides AT, Spyropoulos ND (1991). A survey on supernumerary molars. Quintessence Int.

[8] Menardía-Pejuan V, Berini-Aytés L, Gay-Escoda C (2000). Supernumerary molars. A review of 53 cases. Bull Group Int Rech Sci Stomatol Odontol.

[9] Açıkgöz A, Açıkgöz G, Tunga U, Otan F (2006). Characteristics and prevalence of non-syndrome multiple supernumerary teeth: a retrospective study. Dentomaxillofac Radiol.

[10] Leco Berrocal MI, Martín Morales JF, Martínez González JM (2007). An observational study of the frequency of supernumerary teeth in a population of 2000 patients. Med Oral Patol Oral Cir Bucal.

[11] Harris EF, Clark LL (2008). An epidemiological study of hyperdontia in American blacks and whites. Angle Orthod.

[12] Yagüe-García J, Berini-Aytés L, Gay-Escoda C (2009). Multiple supernumerary teeth not associated with complex syndromes: a retrospective study. Med Oral Patol Oral Cir Bucal.

[13] Gündüz K, Celenk P, Zengin Z, Sümer P (2008). Mesiodens: a radiographic study in children. J Oral Sci.

[14] Miloglu O, Cakici F, Caglayan F, Yilmaz AB, Demirkaya F (2010). The prevalence of root dilacerations in a Turkish population. Med Oral Patol Oral Cir Bucal.

[15] Goya HA, Tanaka S, Maeda T, Akimoto Y (2008). An orthopantomographic study of hypodontia in permanent teeth of Japanese pediatric patients. J Oral Sci.

[16] Peker I, Kaya E, Darendeliler-Yaman S (2009). Clinic and radiographical evaluation of non-syndromic hypodontia and hyperdontia in permanent dentition. Med Oral Patol Oral Cir Bucal.

[17] Gupta SK, Saxena P, Jain S, Jain D (2011). Prevalence and distribution of selected developmental dental anomalies in an Indian population. J Oral Sci.

[18] Afify AR, Zawawi KH (2012). The prevalence of dental anomalies in the Western region of saudi arabia. ISRN Dent.

[19] Patil S, Doni B, Kaswan S, Rahman F (2013). Prevalence of dental anomalies in Indian population. J Clin Exp Dent.

[20] Polder BJ, Van't Hof MA, Van der Linden FP, Kuijpers-Jagtman AM (2004). A meta-analysis of the prevalence of dental agenesis of permanent teeth. Community Dent Oral Epidemiol.

[21] Järvinen S, Lehtinen L (1981). Supernumerary and congenitally missing primary teeth in Finnish children: an epidemiologic study. Acta Odontol Scand.

[22] Ezoddini AF, Sheikhha MH, Ahmadi H (2007). Prevalence of dental developmental anomalies: a radiographic study. Community Dent Health.

[23] Chrcanovic BR, Abreu MH, Custódio AL (2010). Prevalence of enamel pearls in teeth from a human teeth bank. J Oral Sci.

[24] Arys A, Dourov N (1987). Enamel pearls in the deciduous teeth. J Biol Buccale.

[25] MacDonald-Jankowski DS, Li TT (1993). Taurodontism in a young adult Chinese population. Dentomaxillofac Radiol.

[26] Garcés-Ortíz LM, Salcido-García JF, Hernández-Flores F, Garcés-Ortíz M (2012). Multiple supernumeraries in a non-syndromic patient. J Clin Pediatr Dent.

[27] Silva AR, Carlos-Bregni R, Vargas PA, Almeida OP, Lopes MA (2009). Peripheral developing odontoma in newborn. Report of two cases and literature review. Med Oral Patol Oral Cir Bucal.

[28] Waingade M, Gawande P, Aditya A, Medikeri RS (2014). Pindborg tumor arising in association with an impacted supernumerary tooth in the anterior maxilla. J Mich Dent Assoc.

